# A phosphoserine phosphatase variant present in the brain of Alzheimer's disease patients favors nuclear mistargeting

**DOI:** 10.1111/febs.70169

**Published:** 2025-07-18

**Authors:** Silvia Sacchi, Valeria Buoli Comani, Ivan Arisi, Francesco Marchesani, Valentina Rabattoni, Omar De Bei, Zoraide Motta, Alessio Peracchi, Stefano Bruno, Loredano Pollegioni, Barbara Campanini

**Affiliations:** ^1^ The Protein Factory 2.0, Department of Biotechnology and Life Sciences University of Insubria Varese Italy; ^2^ Department of Food and Drug University of Parma Italy; ^3^ European Brain Research Institute (EBRI) Rita Levi‐Montalcini Rome Italy; ^4^ Institute of Translational Pharmacology National Research Council Rome Italy; ^5^ Department of Medicine and Surgery University of Parma Italy; ^6^ Department of Chemistry, Life Sciences and Environmental Sustainability University of Parma Italy

**Keywords:** l‐serine, moonlighting function, phosphorylated pathway, serine metabolism, serinosome

## Abstract

Phosphoserine phosphatase (PSP) catalyzes the dephosphorylation of 3‐phosphoserine, which is the final step in the *de novo* biosynthesis of l‐serine (l‐Ser) via the phosphorylated pathway in human astrocytes. Individuals who are homozygous or compound heterozygous for functionally defective PSP variants exhibit reduced cerebrospinal fluid l‐Ser levels and severe neurological symptoms. In the present study, single nucleotide polymorphisms in PSP were identified in hippocampal samples from Alzheimer's disease (AD) patients. Two single nucleotide polymorphisms, likely forming a haplotype (chr7:56088825 T>A and chr7:56088811 T>C, encoding R27S and D32G PSP variants, respectively), were detected exclusively in AD patients (three females and one male). Biochemical characterization of the recombinant R27S/D32G PSP enzyme revealed a slight decrease in thermostability, a 38‐fold reduction in catalytic efficiency and a two‐fold increase in IC_50_ for l‐Ser, with the D32G substitution being the primary contributor to these effects. Despite its reduced enzyme activity, the R27S/D32G variant did not impair l‐Ser biosynthesis either in an *in vitro* reconstructed pathway or in U251 human glioblastoma cells ectopically expressing the variant under heterozygous conditions. In these cells, PSP colocalized extensively with the other two phosphorylated pathway enzymes, namely phosphoglycerate dehydrogenase and phosphoserine aminotransferase, suggesting that they assemble into a functional complex, known as the serinosome. Notably, the R27S/D32G PSP variant exhibited increased nuclear localization compared to the wild‐type enzyme. This mislocalization raises the intriguing possibility that PSP's moonlighting functions, including its putative role as a protein phosphatase, may be affected, potentially implicating it in pathways beyond l‐Ser biosynthesis.

## Graphical abstract

l‐serine levels in the human brain are maintained by the phosphorylated pathway, a three‐step process catalyzed by phosphoglycerate dehydrogenase (PHGDH), phosphoserine aminotransferase (PSAT) and phosphoserine phosphatase (PSP). These enzymes assemble in a metabolon called the serinosome. The double R27S/D32G PSP variant was identified in the brain of Alzheimer's disease patients and showed reduced catalytic activity and altered subcellular localization.

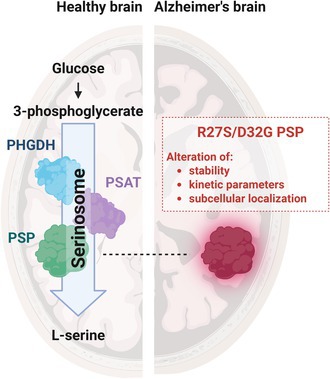

**Abbreviations**
3‐PG: 3‐phosphoglycerate
3‐PS: 3‐phospho‐l‐serine
AD: Alzheimer's disease
AF3: AlphaFold 3
CD: circular dichroism
d‐Ser: d‐serine
Gly: glycine
l‐Glu: l‐glutamate
l‐Ser: l‐serine
NLS: nuclear localization sequence
NMDAR: N‐methyl‐D‐aspartate receptor
PDB: Protein Data Bank
PHGDH: phosphoglycerate dehydrogenase
pLDDT: predicted local distance difference test
PNP: phosphonucleotide phosphorylase
PP: phosphorylated pathway
PSAT: phosphoserine aminotransferase
PSP: phosphoserine phosphatase
rmsd: root‐mean‐square deviation
ROI: regions of interest
SAT: serine acetyltransferase
SDD: serine deficiency disorder
SEC: size‐exclusion chromatography
SNP: single nucleotide polymorphism
TBS: Tris‐buffered saline
TCA: trichloroacetic acid
*T*_m_
: melting temperature
wt: wild‐type

## Introduction

**Fig. 1:**
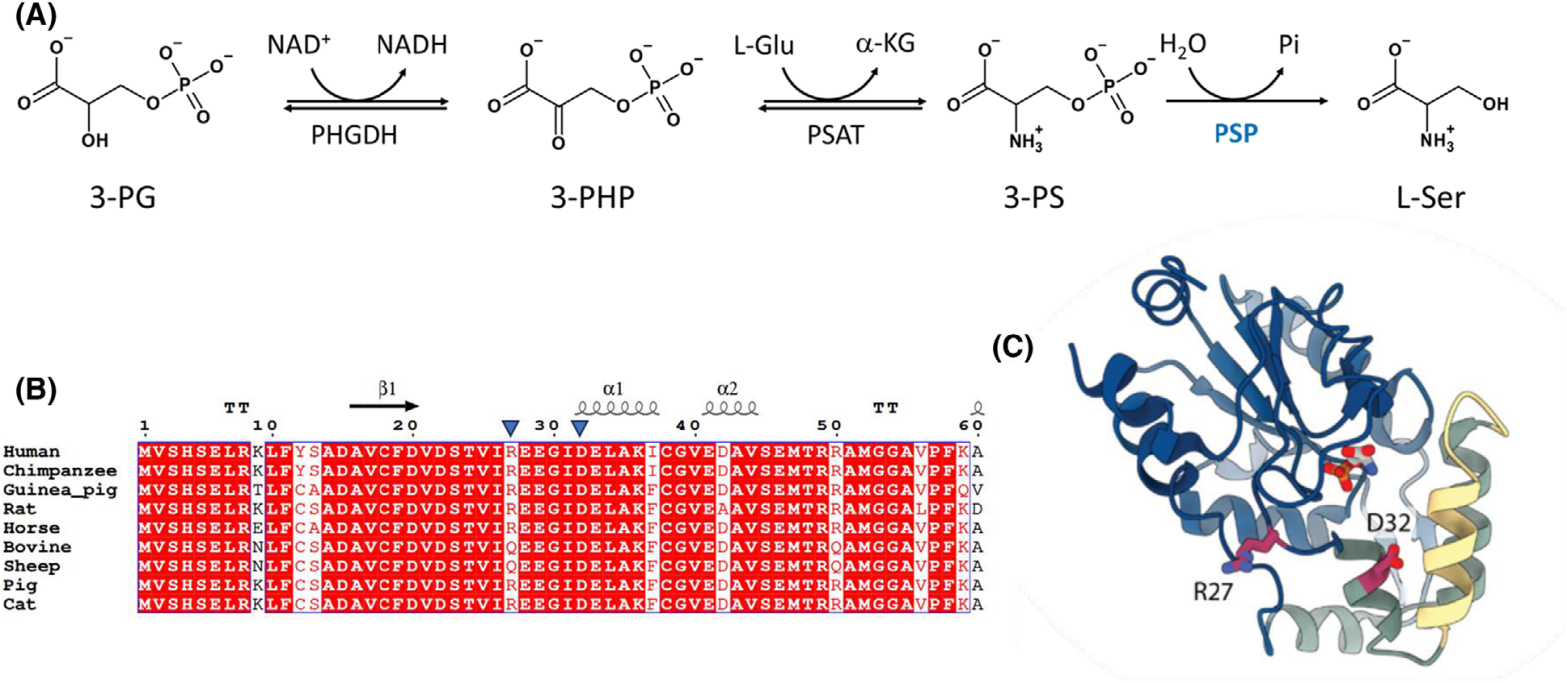
PSP and the phosphorylated pathway. (A) The phosphorylated pathway (PP) is a three‐step pathway, formed by 3‐phosphoglycerate dehydrogenase (PHGDH), phosphoserine aminotransferase (PSAT) and phosphoserine phosphatase (PSP), that catalyzes the conversion of the glycolytic intermediate 3‐PG to l‐Ser. (B) Multiple sequence alignment of mammalian PSP sequences. Sequences were retrieved from UniProt, aligned with the UniProt align tool with default parameters. Secondary structure elements above the sequence are taken from PDB ID 1L8O. The positions of R27 and D32 are marked with blue triangles above the sequence. Visualization was performed using espript [2]. UniProtKB accession numbers are as follows: P78330 *Homo sapiens*, H2QUL8 *Pan troglodytes*, H0VWZ9 *Cavia porcellus*, Q5M819 *Rattus norvegicus*, F6UUR3 *Equus caballus*, Q2KHU0 *Bos taurus*, W5PJN0 *Ovis aries*, F1RIU1 *Sus scrofa*, M3WT54 *Felis catus*. (C) The three‐dimensional structure of human PSP (PDB ID 1L8O, chain B) is shown in ribbon mode with the enzyme's products (l‐Ser and phosphate) displayed as sticks within the active site, whereas the residues corresponding to the variants under investigation are highlighted in purple sticks. TT stands for ‘turns’. The Rossmann‐like domain is shown in blue, the subdomain is shown in green and the flexible helix–loop region (residues 40–56) is shown in yellow. Visualization was performed using chimera x.

The local biosynthesis of l‐serine (l‐Ser) in the human brain is essential for maintaining adequate concentrations of the amino acid, which is the precursor of important metabolites such as one‐carbon unit donors, cysteine and phospholipids, as well as of two neuroactive signaling molecules, glycine (Gly) and d‐serine (d‐Ser) [1]. The three‐step pathway for l‐Ser biosynthesis (the phosphorylated pathway, PP) (Fig. 1A) starts from the glycolytic intermediate 3‐phosphoglycerate (3‐PG), which is converted into 3‐phosphohydroxypyruvate by phosphoglycerate dehydrogenase (PHGDH) [3], followed by the transamination of 3‐phosphohydroxypyruvate to 3‐phospho‐l‐serine (3‐PS) catalyzed by phosphoserine aminotransferase (PSAT) [4]. The last irreversible step is a dephosphorylation reaction catalyzed by phosphoserine phosphatase (PSP) [1, 5].

Human PSP is a homodimeric enzyme that catalyzes the dephosphorylation of 3‐PS via a typical split phosphoaspartyl transferase mechanism, with the transient transfer of the phosphate group to an aspartate residue in the active site [6]. The enzyme activity depends specifically on Mg^2+^ ions and is inhibited by Ca^2+^ ions [7]. l‐Ser, the final product of the PP, acts as an inhibitor of isolated PSP (IC_50_ ~300 μm) [5]. However, physiological levels of l‐Ser do not appreciably affect the metabolic flux when the entire pathway is reconstructed [8]. This suggests that, in the human brain, l‐Ser regulates its own biosynthesis essentially through the activation of the PK2 isoform of pyruvate kinase [9].

We have recently demonstrated that in human differentiated astrocytes the three enzymes of the PP are clustered within a distance of 40 nm. This proximity is compatible with the formation of a transient metabolon that we have named ‘serinosome’ [8], for which the inferred regulatory function is still under investigation.

The casebook of serine deficiency disorders (SDDs) and Neu‐Laxova syndrome, a serious form of SDD associated with perinatal‐death, is populated by multiple cases of inactivating mutations in the genes encoding PHGDH and PSAT [10, 11, 12]. Variants with lower activity are usually associated with more serious conditions, supporting the idea that SDDs encompass a spectrum of phenotypes. On the other hand, PSP variants accounting for SDDs are fewer, and are usually associated with very severe conditions [13, 14, 15], suggesting that a moderate decrease in the enzyme's activity is unlikely to cause highly evident symptoms. This further suggests that substitutions causing only a slight reduction in enzymatic activity may have gone undetected and could be associated with underdiagnosed conditions. Moreover, the possibility that slightly hypofunctional variants may alter the serinosome assembly adds another level of complexity to the molecular mechanisms underlying the development of SDDs. Recently, PSP variants associated with milder conditions have been identified, with eight of them associated with oligoasthenozoospermia [16]. For example, our groups characterized a new PSP variant (N133S) associated with a neurodevelopmental syndrome and myelopathy that showed reduced stability and a misassembly of the serinosome [17].

In the present study, we have identified two PSP variants (R27S and D32G) (Fig. 1B,C) which likely form a haplotype in brain tissues from Alzheimer's disease (AD) patients. We performed a deep genetic, biochemical and cellular characterization. Our overreaching aim was to shed light on the various genetic alterations in the PP enzymes related to different pathological conditions, with the final goal of envisaging targeted therapeutic interventions.

## Results

### Identification of R27S and D32G PSP variants

Four single nucleotide polymorphisms (SNPs) in PSP‐encoding gene were identified only in hippocampus samples from AD subjects (*n* = 11) and not in healthy controls (*n* = 10) (Table S1): two substitutions (rs78599516 and rs74445297) were missense and two (rs202027697 and rs199851385) synonymous (Table S1). Most of the SNPs were identified in female subjects: the variants were found in four subjects (three females and one male) and the missense ones in three of these subjects (two females and one male). Two female subjects carried all four SNPs, the male subject carried both missense variants and one female subject carried only the rs78599516 SNP.

**Fig. 2:**
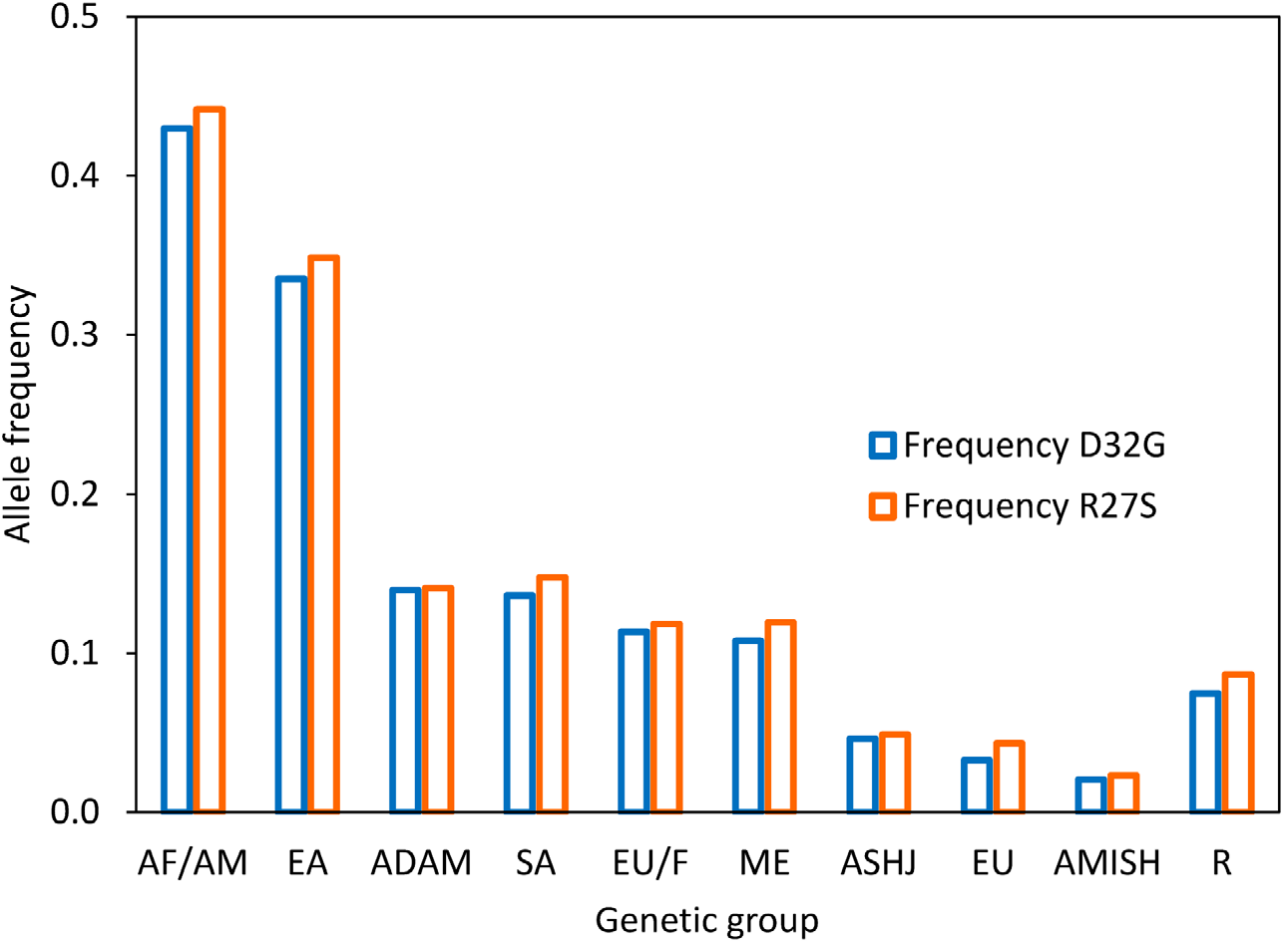
Population frequencies of the 7‐56 088 811‐T‐C (D32G) and 7‐56 088 825‐T‐A (R27S) variants as published in gnomAD ver 4.1.0 (exomes + genomes). ADAM, Admixed American; AF/AM, African/African American; AMISH, Amish; ASHJ, Ashkenazi Jewish; EA, East Asian; EU/F, European (Finnish); EU, European (non‐Finnish); ME, Middle East; R, remaining; SA, South Asian. Total allele frequency is as follows: D32G: 0.0687 (GRCh38); R27S: 0.0798 (GRCh38).

The two missense variants code for the D32G and R27S substitutions in the PSP sequence (Fig. 1 and Table S1) and were consistently found in heterozygosis. Although D32 is a strictly conserved residue, position 27 tolerates conservative substitutions (Fig. 1B). Both D32G and R27S PSP variants are listed in ClinVar (NM_004577.4(PSPH):c.95A>G (p.Asp32Gly) and NM_004577.4(PSPH):c.81A>T (p.Arg27Ser)) and are classified as benign/likely benign (Table 1). Different datasets are available on gnomAD, leveraging on GRCh38 and GRCh37. Analysis using gnomAD v2.1.1 (genome assembly GRCh37) and gnomAD version v4.1.0 (genome assembly GRCh38) indicated that both variants are relatively frequent in the population, with a total allele frequency of 0.06 or higher, which increases shifting from gnomAD v2.1.1 to v4.1.0. The population frequency of the two variants varies greatly, being prevalent in Africans/African Americans and East Asians (Fig. 2). In Africans/African Americans, the allele frequency reaches and surpasses a surprising 0.4. Even more interestingly, the ‘variant co‐occurrence’ function in gnomAD (only available for gnomAD v2.1.1) reasonably supports that the two variants never appear in isolation but rather form a haplotype (the probability that these two variants occur in different haplotypes is < 1%, based on the probability distribution used in gnomAD). In gnomAD v4.1.0, the database records include the variant impact on splicing and protein according to different *in silico* prediction tools, including PolyPhen and the recent Pangolin, that classify D32G as possibly damaging or deleterious (Table 1).

**Table 1:** Information retrieved from ClinVar and gnomAD (v4.1.0) databases on D32G and R27S PSP variants. Scores obtained by different *in silico* precision tools are reported, together with a color‐code (from blue to red based on predicted severity) to represent the impact of the substitution on protein function.

Variant	Clinical significance (ClinVar)	cadd	revel	spliceai	pangolin	phylop	polyphen
D32G	Benign/likely benign	31.0	0.692	0.990	0.900	4.74	0.960
R27S	Benign	13.8	0.461	0.00	0.00	−0.440	0.00700

The results of Cochran–Mantel–Haenszel test across the 10 populations in the most recent gnomAD v4.1.0 (χ^2^ = 1044.6, df = 1, *P* < 2.2 × 10^–16^, common OR = 0.8446, CI common OR = 0.8360–0.8533) show that the two variants do not have exactly the same dichotomic distribution across the different ethnic groups. In more detail, post‐hoc Fisher's exact tests (false discovery rate < 0.05) show that in the following groups the two variants do not have the same distribution: African/African American, East Asian, South Asian, European (Finnish), European (non‐Finnish) and remaining. Nevertheless, the general observation of R27S frequency being similar to D32G one applies equally to the 10 populations because the large sample size (tens of thousands subjects in each population) implies that even tiny differences between the two variants may lead to significant statistical testing, although the effective odds ratio (a metric for the biological effect to be detected) is always close to 1.0 (0.907 ± 0.069). This observation, coupled to the very low probability that these two variants occur in different haplotypes, supports the hypothesis that the two variants belong to the same haplotype.

### Biochemical characterization of PSP variants

**Fig. 3:**
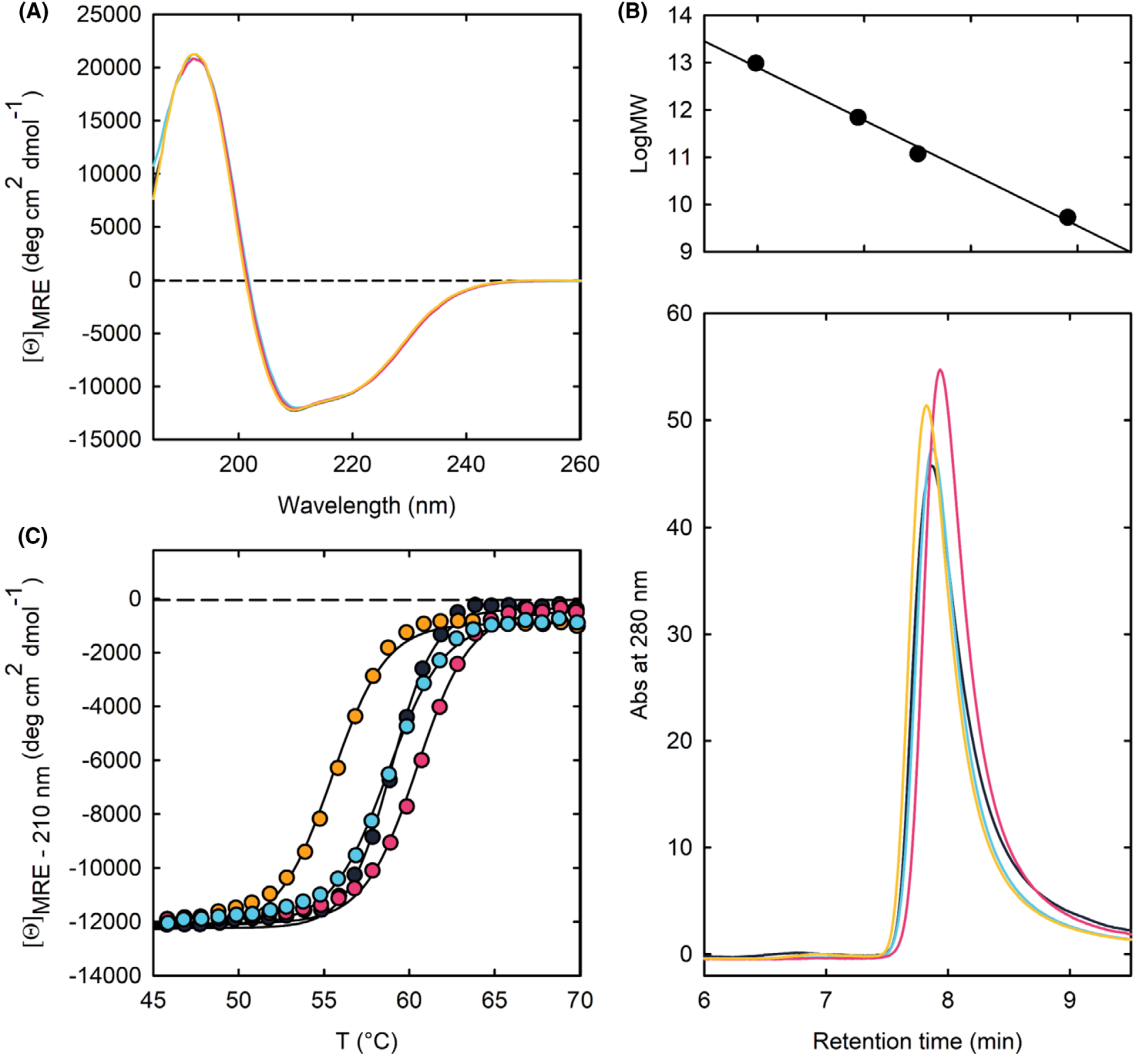
Biophysical characterization of PSP variants (wt, black; D32G, pink; R27S, blue; D32G/R27S, orange). (A) CD spectra collected in buffer P at 20 °C, *n* = 3 technical replicates. (B) Oligomeric state characterization of PSP variants by SEC. Upper: calibration line for molecular mass determination, covering a range from 440 to 17 kDa. The reference data include, from left to right, ferritin (440 kDa), alcohol dehydrogenase (140 kDa), conalbumin (75 kDa) and myoglobin (17 kDa). Lower: SEC chromatograms obtained from an Agilent AdvanceBio 300 Å column. The proteins were analyzed at a concentration of 2 mg·mL^−1^. Data shown is a representative run of two independent experiments. (C) Thermal denaturation curves collected in buffer P at 210 nm for wt and variants of PSP, *n* = 1.

Three variants of PSP were produced recombinantly in *Escherichia coli*: (a) the variants carrying the single substitutions D32G or (b) R27S, and (c) the variant carrying the double substitution (D32G/R27S) to account for the reported co‐occurrence of these substitutions. The PSP variants were expressed as soluble proteins and purified to homogeneity (> 90% pure) with good yields (16 mg·L^−1^ for R27S, 28 mg·L^−1^ for D32G and 20 mg·L^−1^ for R27S/D32G PSP). wtPSP was also expressed and purified and used for comparison. The circular dichroism (CD) spectra of the wild‐type (wt), single and double substituted PSP variants were largely superimposable (Fig. 3A). Likewise, the substitutions did not seem to affect the quaternary structure of the enzyme, as assessed by size‐exclusion chromatography (SEC) (Fig. 3B). Indeed, the apparent molecular mass at 2 mg·mL^−1^ protein concentration was 53, 52, 49 and 57 kDa for the wt, R27S, D32G and R27S/D32G PSP variants, respectively, in excellent agreement with the expected molecular mass of the dimeric form of the protein (54 kDa). The estimated mass did not change when the protein concentration was 20‐fold lower (Table 2). However, although the R27S substitution did not affect the size of the protein, the D32G one resulted in a slight decrease in the apparent molecular mass, as well as in a slight increase when in combination with the R27S substitution.

**Table 2:** Functional and biophysical properties of PSP variants. *T*_m_ was calculated from the thermal denaturation curves followed at 210 nm in the presence of 0.125 mm MgCl_2_ (Fig. 3C). The IC_50_ for l‐Ser was measured at a concentration of 3‐PS equal to the *K*_m_. *v*_o_ and *v*_*i*_ are the initial rates calculated using Eqns (2) and (3) at the physiological concentration of 3‐PS (5 μm) in the absence and presence of 1 mm l‐Ser, respectively. Molecular mass (MW) values were calculated in two independent runs at 0.1 and 2 mg·mL^−1^ protein concentration.

	*T*_m_ (°C)	*k*_cat_ (s^−1^)	*K*_m_ (μm)	*k*_cat_/*K*_m_ (m ^−1^·s^−1^)	IC_50_ l‐Ser (μm)	*v*_0_/*E*_t_ (s^−1^)	*v*_*i*_ /*E*_t_ (s^−1^)	MW (kDa)
wt	59 ± 1	38 ± 3	42 ± 12	0.90 × 10^6^	273 ± 31	4.05	0.88	53 ± 1
R27S	58 ± 1	39 ± 1	50 ± 3	0.78 × 10^6^	171 ± 15	3.54	0.52	52 ± 1
D32G	61 ± 1	2.3 ± 0.1	53 ± 5	4.34 × 10^4^	746 ± 26	0.198	0.085	49 ± 1
R27S/D32G	56 ± 1	2.2 ± 0.1	75 ± 12	2.93 × 10^4^	523 ± 74	0.138	0.047	57 ± 1

The thermal stability of the variants was assessed by measuring the CD signal at 210 nm as a function of temperature in the presence of 0.125 mm magnesium chloride, a concentration within the physiological range (Fig. 3C). The melting temperature (*T*_m_) calculated by fitting the melting curves to Eqn (1) did not differ substantially for the single variants D32G and R27S PSP compared to wt. On the other hand, the *T*_m_ value calculated for the double variant was reduced by approximately 3.4 °C (Fig. 3C and Table 2), suggesting that only the co‐presence of both the substitutions affects PSP thermal stability. Notably, a similar *T*_m_ was estimated for the A35T PSP variant, which was identified in a Pakistani family affected by intellectual disability [5].

**Fig. 4:**
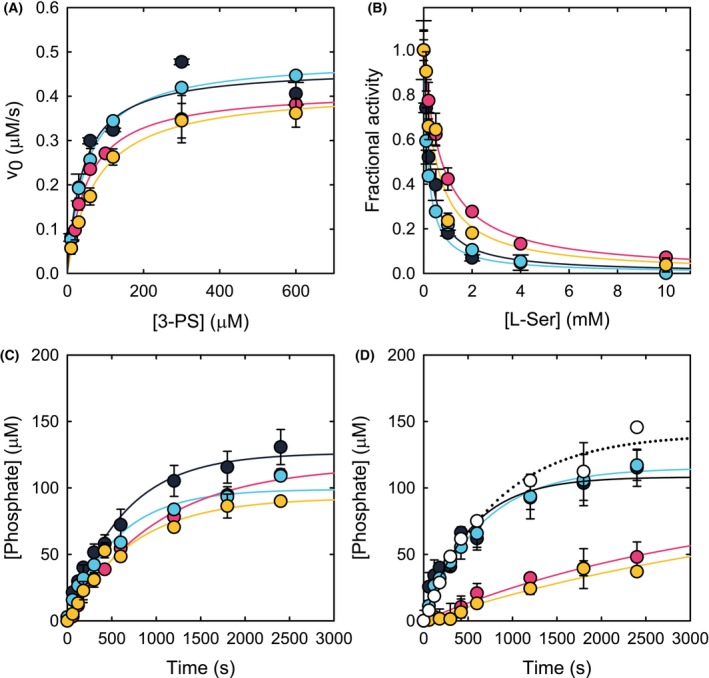
Functional characterization of PSP variants (wt, black dots; D32G, pink dots; R27S, blue dots; D32G/R27S, orange dots). (A) Dependence of the initial velocity of wt PSP and variants on 3‐PS concentration. Different enzyme concentrations were used (wt: 12 nm; D32G: 180 nm; R27S: 12 nm; R27S/D32G: 180 nm). Lines through data points are the fitting to Eqn (2) with *K*_m_ and *k*_cat_ as reported in Table 2. (B) Dependence of the relative initial velocity of wt PSP and variants on l‐Ser concentration. Data were collected at a 3‐PS concentration equal to the *K*_m_ value. Lines through data points are the fitting to Eqn (3) with IC_50_s as reported in Table 2. (C) Kinetics of the *in vitro* reconstructed PP in the absence of l‐Ser. The concentration of enzymes matches that measured in human astrocytes: 0.82 μm PHGDH, 1.14 μm PSAT and 0.12 μm PSP. (D) Kinetics of the *in vitro* reconstructed PP in the presence of 1 mm l‐Ser. The concentration of enzymes matches that measured in human astrocytes: 0.82 μm PHGDH, 1.14 μm PSAT and 0.12 μm PSP. Open dots represent a 1 : 1 mixture of wt (0.06 μm) and R27S/D32G (0.06 μm) PSP; the dotted line represents the fitted curve of these points. Data represent the average of two independent experiments; error bars represent the SD.

The kinetic parameters were determined in the presence of 3 mm MgCl_2_, a saturating concentration for the wt PSP [5] (Fig. 4A and Table 2). l‐Ser is a known inhibitor of PSP and primarily affects poorly active variants [5]. The IC_50_ for l‐Ser was determined at a concentration of 3‐PS equal to the *K*_m_ (Fig. 4B and Table 2). The R27S substitution did not markedly alter neither the catalytic parameters of the enzyme, nor the IC_50_ for l‐Ser, in line with the observation that R27 in PSP tolerates the substitution with polar residues, such as serine (Fig. 1). On the other hand, the substitution of the strictly conserved D32 residue resulted in a severe impairment of the catalytic activity, mainly affecting the *k*_cat_, which decreased by approximately 20‐fold. This was reflected also in a 26‐fold reduction of the catalytic efficiency (*k*_cat_/*K*_m_). Considering that the 3‐PS cellular concentration has been estimated to be around 5 μm (i.e. well below the *K*_m_), the enzyme works under *k*_cat_/*K*_m_ regime in the cell, and the effect of the substitution on the activity is expected to be severe. Interestingly, as already observed for other pathogenic PSP variants [5], the reaction product affected D32G activity to a lower extent compared to wt PSP: the IC_50_ for l‐Ser of this variant was appreciably higher than the one for the wt (~750 vs. ~270 μm, respectively). A similar effect was observed for the R27S/D32G double variant (Table 2). Accordingly, the increase in IC_50_ for l‐Ser might partially compensate for the reduced catalytic activity *in vivo*, where l‐Ser has a concentration of approximately 1 mm [8, 18, 19]. The calculated rates at 5 μm 3‐PS and 1 mm l‐Ser are reported in Table 2. These values indicate that, although the variants exhibit lower susceptibility to inhibition by l‐Ser, this effect is insufficient to effectively compensate for the decrease in catalytic efficiency. Noteworthy, the kinetic properties associated with the D32G substitution dominate the double variant that shows a reduced *k*_cat_, a decreased catalytic efficiency and an increased IC_50_ for l‐Ser. Indeed, D32 and R27 are residues located outside the active site, and their primary effect may be on protein dynamics rather than on substrate binding.

The effect of substitutions was also studied on the overall *in vitro* reconstructed PP [8], to better mimic physiological conditions. The flux through the pathway was measured as the amount of phosphate produced over time in the presence of pseudo‐physiological concentrations of substrates and products (Fig. 4), either in the absence (Fig. 4C) or presence (Fig. 4D) of 1 mm l‐Ser. In the absence of l‐Ser, the rate of phosphate accumulation was very similar for variants and wt PSP, albeit a slight reduction was more pronounced for D32G and R27S/D32G PSP (Fig. 4C). Conversely, at 1 mm l‐Ser, phosphate production was markedly reduced in D32G and R27S/D32G PSP (Fig. 4D), as observed in other low‐activity PSP variants linked to severe neurological disorders [8]. Notably, the lower sensitivity of D32G and R27S/D32G variants to l‐Ser inhibition did compensate for their reduced catalytic activity, which is thus responsible for the large decrease in the rate of l‐Ser production.

Being the R27S/D32G substitutions present in heterozygosis, this condition was simulated by measuring the flux through the PP in the presence of identical amounts of both the wt and R27S/D32G PSP (halving their singular concentration), in the presence of 1 mm l‐Ser (Fig. 4D, open dots). The kinetic trace obtained was superimposable to that of the wt PSP alone, further confirming that PSP is not rate limiting in the PP and a 50% decrease in fully active enzyme does not affect the metabolic flux to l‐Ser.

### *In silico* study of the effect of substitutions on protein structure and dynamics

**Fig. 5:**
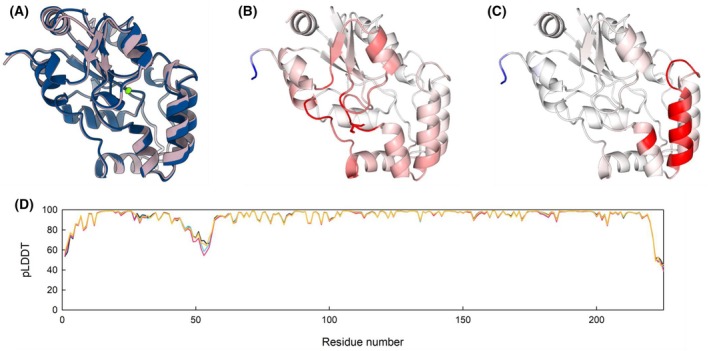
Prediction of the effect of the D32G and R27S substitutions on the structure and dynamics of PSP. (A) Superimposition of alphafold3 predicted structures of wt (dark blue) and R27S/D32G (pink) PSP. Because PSP is a magnesium‐dependent enzyme, the ion was included in the prediction and is shown as a green sphere. dynamut evaluation of changes in vibrational entropy upon substitution of (B) Arg27 with Ser or (C) Asp32 with Gly. Rigidification is shown in blue, and increased flexibility is shown in red. (D) pLDDT per residue for wt PSP (black line), R27S (blue line), D32G (pink line) and R27S/D32G (orange line). Visualization was performed using chimera x.

Structural predictions of R27S, D32G and R27S/D32G variants, were generated using AlphaFold 3 (AF3) (Fig. 5) [20]. Additionally, the wt protein structure was also predicted using AF3 for comparison with the available Protein Data Bank (PDB) model (PDB ID 1L8O). All predictions displayed global predicted local distance difference test (pLDDT) scores sufficiently high to be considered reliable. Furthermore, the root‐mean‐square deviation (rmsd) on alpha carbons between the experimental structure and the AF3 prediction of wt PSP was very low (0.479 Å), confirming the high similarity of the superimposed structures. These controls provide validation for subsequent structural comparisons.

The predicted AF3 structures for wt and substituted PSP protein variants were largely superimposable, with alpha carbon rmsd (Cα‐rmsd) values below 1 Å (Fig. 5A). Although the overall predictions were robust, all predicted structures exhibited lower pLDDT scores in the region encompassing residues 45–56 (Fig. 5D). This may reflect a reduced predictive accuracy in this region, but it could also indicate an intrinsic flexibility that might explain the observed pLDDT values. These differences may be absent at the 3D structural level, or it is possible that the advancements in AF3 are still insufficient to overcome the limitations previously identified in alphafold regarding the prediction of structural effects induced by single substitutions [21].

To delve deeper into the findings obtained with AF3 and to distinguish flexibility from reduced predictive capacity in the flexible subdomain region of PSP, the online tool dynamut was employed to study the effect of substitutions on enzyme dynamics. Figure 5B,C shows a direct comparison of the flexibility of the analyzed variants relative to the wt protein. Although the R27S substitution subtly affects the overall protein dynamics, the D32G substitution locally and strongly affects the flexible helix–loop region. Unfortunately, dynamut does not allow analyses involving more than one substitution at a time.

In summary, although the substitutions do not exhibit pronounced effects on the overall structure of PSP, they appear to affect the protein local flexibility in a region that is critical for enzymatic function.

### Cellular distribution of PP enzymes

U251 human glioblastoma cells were selected as a model cell system to mimic a heterozygous condition because of the documented endogenous expressions of the PP enzymes (15.28 ± 1.91, 3.06 ± 0.38 and 3.63 ± 1.34 ng per 10^5^ cells for PHGDH, PSAT and PSP, respectively) [22]. The R27S/D32G PSP variant and the wt enzyme (as a positive control) were overexpressed in the U251 human glioblastoma cell line as chimeric proteins containing a C‐terminal AviTAG for detecting ectopic PSP protein variant expression. Bands corresponding to the endogenous protein and the ectopically expressed PSP variants were identified based on the different molecular masses (25 and 27 kDa, respectively, as a result of the presence of the 1.8 kDa AviTAG fused to the latter variants). U251 cell clones transfected with the empty vector (U251^NEG^) were analyzed as negative controls. Densitometric analysis indicated that the endogenous PSP level was 2.29 ± 0.47, 5.78 ± 1.96 and 4.06 ± 1.97 ng per 10^5^ cells for negative controls, wt and R27S/D32G PSP variant transfected cells, respectively. The level of AviTAG‐PSP was 10.84 ± 3.32 and 6.15 ± 1.89 ng per 10^5^ cells for cell clones ectopically expressing wt and R27S/D32G PSP variants, respectively.

**Fig. 6:**
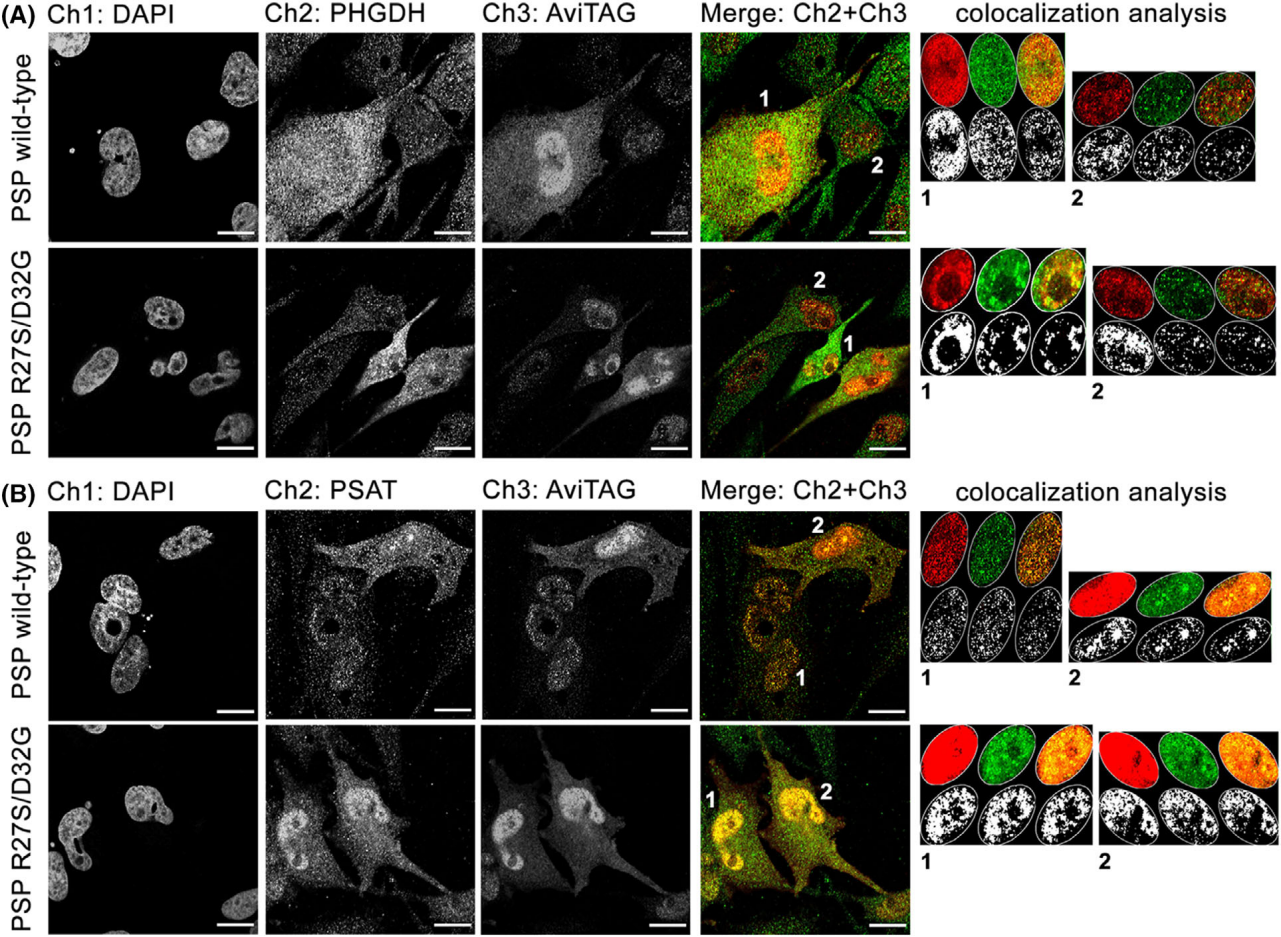
Immunolocalization of the PP's enzymes in U251^+wt^ and U251^+R27S/D32G^ cells as detected by confocal analysis on single sections including nuclei. (A, B) Double staining immunofluorescence analyses were performed by the monoclonal mouse anti‐AviTAG antibody to label the ectopically expressed PSP variants, and the polyclonal rabbit anti‐PHGDH (A) and mouse anti‐PSAT (B, sequential staining). Single‐channel images are shown, along with merged images to illustrate the overlap of green and red signals. Scale bar = 15 μm. The colored panels on the right show details of the colocalization analysis performed within selected ROIs that define the nuclei of the cells numbered in the merge panels (A) and (B). Images are representative of the 10 fields acquired for each coverslip and of the colocalization analysis performed on selected cells (*n* = 30 and 40 for U251^+wt^ and U251^+R27S/D32G^, respectively).

**Fig. 7:**
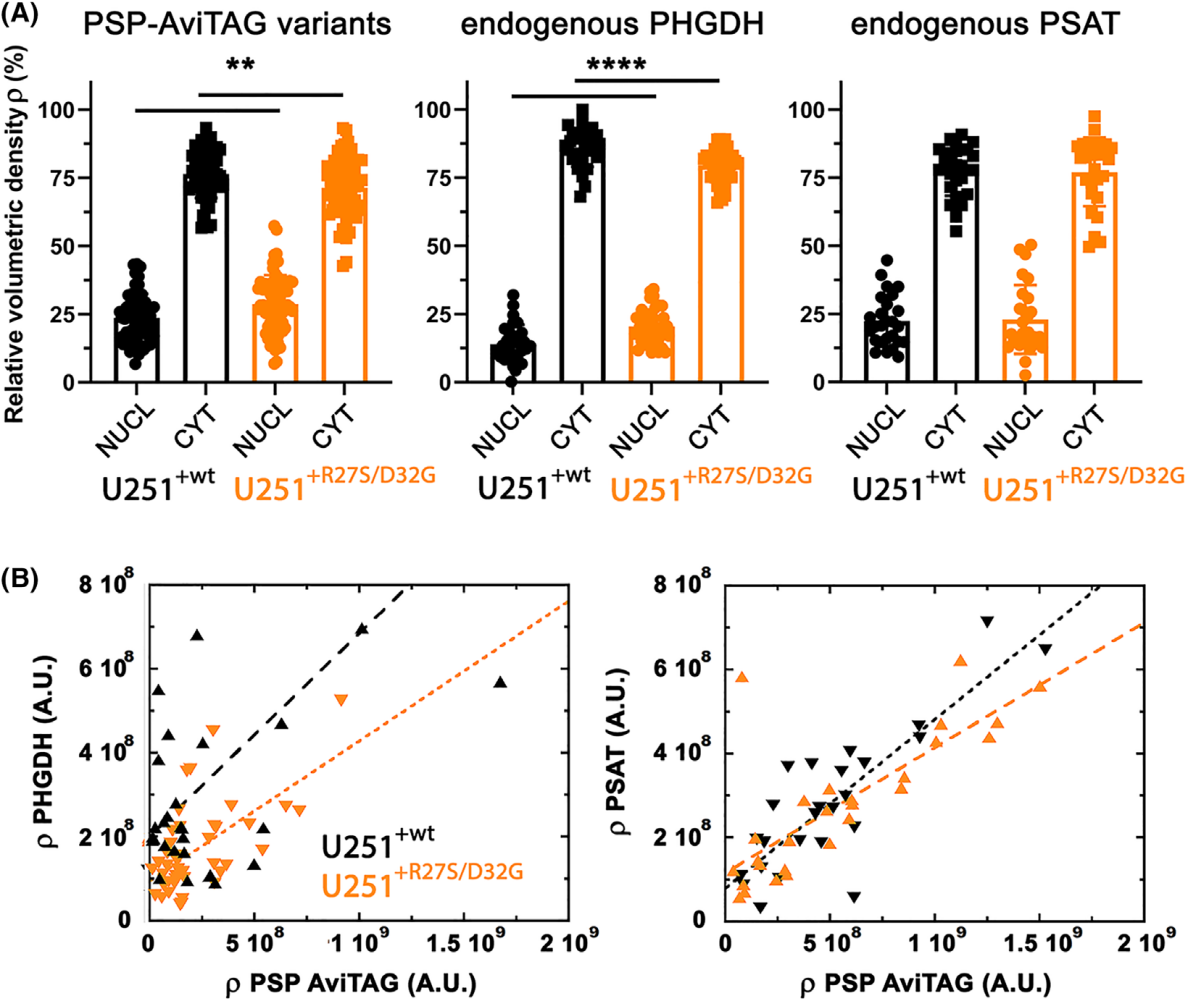
Cellular distribution of the PSP AviTAG protein variants and PP's enzymes in U251^+wt^ (black) and U251^+R27S/D32G^ (orange) cells. (A) Based on confocal analysis and the detected density of the corresponding signals, the fraction of the different proteins showing nuclear vs. cytoplasm localization (expressed as relative volumetric density, ρ) was assessed by normalizing the value of the 2D density of each signal to the volume of the two compartments. Bars represent the mean ± SD, dots indicate values determined for U251^+wt^ and U251^+R27S/D32G^ cells (*n* > 30). Statistical significance between groups was assessed using the non‐parametric Mann–Whitney *U*‐test; **P < 0.01; ****P < 0.0001. (B) Plot showing apparent linear correlation between the levels of overexpressed PSP AviTAG (estimated as above) and the endogenous PHGDH (left) and PSAT (right) ones. Analyses were performed on selected cells (*n* > 30) across different acquisition fields (10 each coverslip).

Double staining immunofluorescence analyses were performed on fixed U251^+wt^ and U251^+R27S/D32G^ cell clones, as well as on the U251^NEG^ control, to investigate the localization of the PSP variants and their relative distribution compared to the endogenously expressed protein partners belonging to the PP (i.e. PHGDH and PSAT). In U251^+wt^ and U251^+R27S/D32G^ cell clones, most cells express the PSP‐AviTAG variants (70% for the wt and 85% for the R27S/D32G variant), although to different extents: distinct cell populations expressing low and high levels of the PSP variants are apparent. The levels of the overexpressed PSP‐AviTAG variants and endogenous PP proteins (expressed as volumetric density, ρ) were evaluated in individual cells by measuring the 2D density of the corresponding signals in the nucleus and the cytoplasm. In U251^+wt^ and U251^+R27S/D32G^ cells, the AviTAG PSP variants, as well as the endogenously expressed PP enzymes, are predominantly cytosolic (Figs 6 and 7A and Table S2), although a minor amount was observed in the nucleus, with a 15–30% nuclear and 70–85% cytoplasmic distribution (Fig. 7A and Table S2). Notably, U251^+R27S/D32G^ cells exhibited a small but statistically significant increase in the nuclear distribution of the overexpressed variant (28% in these cells compared to 23.5% in U251^+wt^ cells; *n* = 68 and 56, respectively, *P* = 0.0067). Immunofluorescence analysis suggested that the cellular levels of endogenous PHGDH and PSAT correlate with those of the overexpressed AviTAG PSP variants: their levels increased in cells showing higher AviTAG signal densities (Fig. 7B). Intriguingly, in U251^+R27S/D32G^ cells, the observed higher nuclear level of the overexpressed PSP variant was mirrored by statistically significant increased levels of endogenous PHGDH: the fraction of PHGDH detected in the nucleus was 20% compared to 13% in U251^+wt^ control cells (*n* = 41 and 30 for U251^+R27S/D32G^ and U251^+wt^ cells, respectively; *P* < 0.0001), whereas no difference in PSAT localization was observed (~20%) (Fig. 7 and Table S2).

Bioinformatic tools for predicting protein subcellular localization suggested a predominantly cytosolic distribution for wt and R27S/D32G PSP variants, as indicated by cello [23] and wolf psort [24]. However, slpred (https://slpred.kansil.org/) indicates a potential for nuclear targeting. Further analysis of the PSP variants' sequence failed to provide conclusive evidence for the presence of a nuclear localization sequence (NLS). Only one potential NLS (position 157–172) was identified by nlstradamus [25] using a two‐ or four‐state static hidden Markov model, but this only using a very low prediction cut off value (0.2). Nonetheless, previous immunolocalization studies showed that the enzymes of the PP are expressed in the U251 cell line (see below) and appear to be partially distributed in the nucleus, especially in the case of PSP [22]. Notably, the PHGDH and PSAT fluorescence signals form dense *punctua* in both cellular compartments. In the U251^+wt^ and U251^+R27S/D32G^ cell lines, the AviTAG signal for ectopically expressed PSP significantly overlapped with the endogenous PHGDH and PSAT ones (Fig. 7), as indicated by the calculated overlap coefficient that measures the co‐occurrence of couple of signals, expressed as overlap (%) in Table 3. This finding suggests that the ectopically overexpressed PSP proteins are engaged in the serinosome, together with the endogenous PP enzymes. In line with recently reported data in human induced pluripotent stem cell‐derived differentiated astrocytes [8], the analysis of signals corresponding to the overexpressed PSP‐AviTAG in U251^+wt^ and U251^+R27S/D32G^ cells (in red) and the endogenous PHGDH or PSAT ones (in green) showed a lower degree of colocalization with PHGDH compared to PSAT (Fig. 6 and Table 3): PHGDH has been previously proposed to be less stably associated to the multi‐enzymatic assembly [8].

**Table 3:** Colocalization analysis of immunofluorescence signals between ectopically expressed PSP variants and PHGDH or PSAT in U251 cells. The reported parameters were determined by using the biop jacop plugin in fiji (imagej). ROIs selected for containing a single cell were cropped during the analysis and independently processed. Cells were divided in three groups based on ectopic expression level of PSP‐AviTAG protein: high, low, no expression (used as negative control). Pearson and Spearman rank coefficients were calculated by applying the Otsu threshold to both the red and green channel. ‘Overlap’ represents the ratio of the area of the AviTAG:PHGDH or AviTAG:PSAT overlapping pixels (yellow) with respect to the total areas of the PHGDH or PSAT pixels (green). Values represent the mean ± SD of results from 10 to 15 imaged U251^+wt^ and U251^+R27S/D32G^ cells.

	Ectopic expression level	Colocalization coefficient					
		Whole cell			Nucleus		
		Pearson	Spearman rank	Overlap (%)	Pearson	Spearman rank	Overlap (%)
PHGDH							
Wild‐type	High	0.41 ± 0.1	0.46 ± 0.15	35 ± 13	0.28 ± 0.1	0.32 ± 0.15	57 ± 17
	Low	0.19 ± 0.06	0.24 ± 0.09	21 ± 12	0.20 ± 0.06	0.19 ± 0.10	24 ± 11
R27S/D32G	High	0.44 ± 0.15	0.50 ± 0.15	33 ± 11	0.38 ± 0.14	0.41 ± 0.17	61 ± 18
	Low	0.20 ± 0.07	0.46 ± 0.60	21.0 ± 6.5	0.23 ± 0.07	0.24 ± 0.13	39.0 ± 13.5
PSAT							
Wild‐type	High	0.67 ± 0.07	0.73 ± 0.08	49.0 ± 7.5	0.69 ± 0.09	0.69 ± 0.10	71 ± 14
	Low	0.66 ± 0.06	0.68 ± 0.09	55.0 ± 8.5	0.69 ± 0.06	0.68 ± 0.11	61 ± 19
R27S/D32G	High	0.66 ± 0.11	0.73 ± 0.08	52 ± 11	0.65 ± 0.14	0.67 ± 0.14	63.5 ± 19.0
	Low	0.68 ± 0.06	0.72 ± 0.06	53 ± 9	0.72 ± 0.07	0.75 ± 0.06	72 ± 9
	No expression	0.10 ± 0.01	0.22 ± 0.05	4.5 ± 2.0	0.05 ± 0.02	0.09 ± 0.05	Below detection

The degree of colocalization was assessed in single cells by comparing the Pearson's and the Spearman's coefficients. The mean values determined for these two parameters were 1.5–3.5‐fold lower for the AviTAG:PHGDH signal pair compared to the AviTAG:PSAT one (Table 3). Worthy of note, in the nucleus of cells expressing high levels of the PSP AviTAG protein variants, a larger colocalization between the overexpressed R27S/D32G PSP variant and PHGDH was detected compared to the wt PSP (Table 3).

The latter finding suggests that the overexpressed R27S/D32G PSP has a higher propensity to be mistargeted to the nucleus than the wt one and likely concurs to the stabilization of the interaction with PHGDH.

### Cellular serine level

**Fig. 8:**
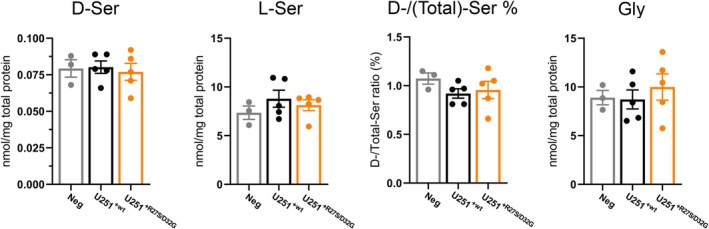
d‐Ser, l‐Ser and Gly levels, and the ratio between d‐/total‐Ser content in U251 cell clones transfected with an empty vector (Neg, gray), U251^+wt^ (black) and U251^+R27S/D32G^ (orange) cells. Bars report the mean ± SEM; dots represent values corresponding to different clones analyzed in at least three technical replicates. Statistical analyses were performed using ordinary one‐way analysis of variance (multiple comparison). In all cases, changes were not statistically significant (*P* > 0.05).

To assess the impact of the PSP R27S/D32G double substitution on intracellular serine and glycine levels, enantiomeric HPLC analyses was performed on U251^+R27S/D32G^, U251^+wt^ cell clones and U251^NEG^ ones as a control. No statistically significant differences were observed in the levels of serine enantiomers or glycine among the groups. However, a slight increase in l‐Ser levels (not reaching a statistical threshold) was apparent in U251^+wt^ clones compared to U251^NEG^ ones, an effect less pronounced in U251^+R27S/D32G^ clones (Fig. 8). The d‐Ser level instead appeared unaffected by the overexpression of wt and PSP variants, resulting in a decrease in d‐Ser over total serine ratio in both groups compared to the negative control.

### Cellular stability

The cellular stability of Avi‐tagged wt and R27S/D32G PSP variants was investigated in U251^+R27S/D32G^, U251^+wt^ cell clones by western blot analysis (Fig. S1). As a control, the levels of endogenous PSP in the same cell clones, as well as in U251^NEG^ cells, were analyzed. Consistently with our previous results [17], PSP is a long‐lived protein. The R27S/D32G PSP variant displayed a reduction in half‐life compared to the wt enzyme: approximately 50% of the variant protein remained after 48 h of cycloheximide treatment, compared to approximately 70% for the overexpressed wt one (Fig. S2). Notably, the N‐terminal AviTAG did not affect protein degradation kinetics because similar time courses were apparent for the endogenous PSP (in control U251 cell clones) and the Avi‐tagged wt PSP.

## Discussion

Subsequent to the first report on a neurometabolic disorder associated with PHGDH and PSP deficiency in three patients [26], many studies have highlighted the association of severe neurological disorders with deficiencies in either one of the three enzymes of the PP in humans [12, 27, 28, 29, 30]. The number of works reporting the involvement of hypoactive enzyme variants in less severe disorders has increased in recent times [17, 31, 32, 33, 34]; nevertheless, our understanding of how substitutions that only modestly impact enzyme activity contribute to the pathogenesis of neurological diseases remains limited.

PSP, which catalyzes the last and the only irreversible step of the PP, is very active with respect to the other two enzymes, with turnover numbers of 45 s^−1^ vs. 3 s^−1^ and 24 s^−1^ for PHGDH and PSAT, respectively. Therefore, in the proposed serinosome, PHGDH is the rate‐limiting step and PSP the driving force to generate l‐Ser, thus bypassing the reversibility of the two previous reactions [8]. Indeed, an up to six‐fold change of PSP concentration around its physiological value does not lead to a significant change in the overall rate of l‐Ser production [8]. On one hand, this makes the pathway more resilient to mutations that decrease the activity of PSP; on the other hand, subtle effects of decreased enzyme activity might go unnoticed.

In the present study, we have identified four SNPs in PSP encoding gene in hippocampus samples from AD subjects only: two (rs78599516 and rs74445297) are missense and two are synonymous. The two missense mutations code for the R27S and D32G substitutions in the amino acid sequence of PSP. Both variants are relatively frequent in the population, with a total allele frequency of 0.06 or higher and are prevalent in Africans/African Americans and East Asians (higher than 0.4). Furthermore, the two variants never appear in isolation, but rather form a haplotype. The double variant characterized in this study shows a dramatically decreased catalytic activity, comparable to that observed in PSP pathogenic variants associated with SDD. The two substitutions affect differently PSP activity and susceptibility to l‐Ser inhibition. The R27S substitution, located in the Rossman‐like domain, does not affect the catalytic activity and has a very limited effect on the IC_50_ for l‐Ser. The D32G substitution, located in the subdomain close to the flexible helix–loop region, has profound effects on activity, with a 20‐fold reduction in catalytic efficiency and a more than three‐fold reduction in l‐Ser inhibition. This substitution is also kinetically dominant on the R27S/D32G double variant, which is less efficient and less prone to inhibition by l‐Ser. The double variant also shows a reduced thermal stability *in vitro*, which is accompanied by a decreased cellular stability. *In silico* predictions support the observed effects of the D32G substitution on enzyme activity, that might be explained by an increased mobility of the helix–loop region affecting the protein dynamics associated to catalysis [35]. Indeed, it is well established that this region plays a crucial role for enzyme activity, being involved in a conformational rearrangement to minimize solvent exposure of catalytic intermediates. This finding is also in agreement with structural effects previously observed on the hypoactive A35T and M52T PSP variants associated with SDD [5]. The *in vitro* reconstructed PP demonstrated that under heterozygous conditions, namely using identical amounts of both wt and R27S/D32G PSP, the kinetic traces were superimposable to the one obtained for the wt PSP alone (Fig. 4). This further confirms that PSP is not rate limiting in the PP and that a 50% decrease in PSP enzymatic activity does not modify the metabolic flux to l‐Ser. Notably, at physiological 1 mm l‐Ser, the PP flux is decreased, but to a lower extent than that expected based on the kinetic parameters (Fig. 4 and Table 2). These findings are mirrored by the results obtained at the cellular level and under heterozygous conditions, with the ectopic expression of the double PSP variant that did not affect to a significant extent the serine enantiomers level, as well as the Gly one. Noteworthy, in the case of PSP inactive variants associated with SDD or NLS, the patients were homozygous or compound heterozygotes for the mutation, whereas, in the case of D32G and R27S/D32G substitutions, the patients are consistently heterozygotes, possibly suggesting a prenatal lethality for homozygous individuals. This observation leads to a stimulating conclusion: because the impact of PSP inactivation on the overall pathway is less pronounced than PHGDH and PSAT inactivation [8], the heterozygotes for poorly active variants might not show any obvious neurological symptoms, but might nevertheless reveal impairment in more subtle functions, such as oligoasthenozoospermia [16] or neurodevelopment [17].

Recent studies on the whole central nervous system [36] demonstrated that among the pathways most significantly altered in AD are the ones related to amino acids metabolism, such as Arg biosynthesis and Ala/Asp/Glu metabolism. AD progression has been associated to Ser metabolism [37] and extracellular levels of l‐ and d‐Ser were reduced at an early stage of AD in the hippocampus of a mouse model characterized by lower glycolytic flux in hippocampal astrocytes [38]. Notably, these pathways show a striking convergence: metabolites related to Arg/Pro metabolism have been proposed to contribute to the dysfunction of N‐methyl‐D‐aspartate receptor (NMDAR) signaling in AD [39, 40], which is also related to the metabolism of the coagonists d‐Ser and Gly (for which levels are linked to the l‐Ser level) and closely linked to energetic metabolism. We recently reported an increase in PHGDH, PSAT and serine racemase levels in AD hippocampal samples [19], in good agreement with the increase determined in brains of individuals affected by AD [41, 42]. We suggested that this could represent a mechanism to increase NMDAR activity by providing more d‐Ser. Although, in healthy men, the increase in d‐Ser/total Ser ratio should represent a way to counteract age‐related cognitive decline mainly related to learning and memory [43], the higher d‐Ser/total Ser ratio levels observed in women during AD onset should make it possible to increase long‐term potentiation. This represents a specific attempt to rescue NMDAR hypofunction by overproducing d‐Ser from serine racemase and the PP.

However, besides subtle perturbations of l‐Ser production induced by the variant (not affecting both Gly and d‐Ser levels), we observed a small but statistically significant effect on cellular localization. In U251^+R27S/D32G^ cells, the PSP‐AviTAG variants are mainly distributed in the cytoplasm, with a minor part in the nucleus. In the U251^+wt^ and U251^+R27S/D32G^ cell lines, independently of the distribution of the ectopically expressed PSP variants, their corresponding signal overlapped with the endogenous PHGDH and PSAT ones, thus suggesting that they are engaged in forming the serinosome. Furthermore, this interaction increases the level of the endogenous PHGDH and PSAT, pointing to a stabilizing effect of the protein–protein interaction involved in the serinosome formation. On average, PHGDH levels are lower in U251^+R27S/D32G^ compared to U251^+wt^ cells, whereas PSAT ones are not affected by the identity of the overexpressed PSP AviTAG protein variant. Notably, in the nucleus, a larger colocalization between the overexpressed R27S/D32G PSP variant and PHGDH was detected compared to the wt PSP: the double substitution apparently favors the nuclear targeting of PSP. In conclusion, at the cellular level, the overexpression of the R27S/D32G PSP variant results in a more pronounced nuclear localization compared to the wild‐type enzyme, which favors colocalization with PHGDH. This finding, along with evidence that overexpression of the R27S/D32G PSP variant only marginally affects l‐Ser content, supports the intriguing hypothesis that mistargeting may influence other moonlighting activities of PSP, potentially linked to its role as a protein phosphatase. On this side, recent studies suggest that PSP may contribute to tumor progression through mechanisms independent of serine biosynthesis [44, 45]. A search using BioGRID, IntAct and STRING identified numerous potential PSP interactors involved in signaling pathways regulated by phosphorylation and dephosphorylation, some of which localize to the nucleus. Although this aspect is beyond the scope of the present study, it warrants further investigation to better understand PSP's role in the pathogenesis of various diseases.

## Materials and methods

### Molecular biology and bioinformatics

The study was designed and conducted following internationally recognized ethical principles for research involving human subjects. Post‐mortem brain samples from the hippocampal CA1 region of AD subjects (*n* = 6 female, *n* = 5 male) and age‐matched control subjects (*n* = 5 female, *n* = 5 male) were obtained from the London Neurodegenerative Diseases Brain Bank (https://www.kcl.ac.uk/neuroscience/facilities/brain‐bank) as described previously [19]. All cases were collected at the Medical Research Council (MRC) London Neurodegenerative Diseases Brain Bank hosted at the Institute of Psychiatry, Psychology and Neuroscience, KCL, under informed consent. The bank operates under a license from the Human Tissue Authority and ethical approval as a research tissue bank (08/MRE09/38 + 5). The whole procedure of targeted Genotyping by Sequencing (Hi‐SNPseq method) to detect SNP variants was performed at CD Genomics (NY, USA), including sample preparation. The harvested DNA was detected by agarose gel electrophoresis and NanoDrop and quantified using a Qubit Fluorometer (Thermo Fisher Scientific, Waltham, MA, USA). In total, 500 ng of DNA per sample was used as input material. Quantified genomic DNA was fragmented using g‐TUBE devices (Covaris, Woburn, MA, USA) and subsequently repaired by treating the sample with a DNA‐damage repair and A‐tailing mix. Adapters incorporating a unique index were ligated to each end. Then, pre‐PCR for whole genome library amplification was performed using a custom panel for target enrichment. The library quality was analyzed by Qubit and real‐time PCR, and average fragment size was estimated using the Agilent 2100 Bioanalyzer (Agilent, Santa Clara, CA, USA). The library was sequenced using the Novaseq PE150 platform (Illumina Corp., san Diego, CA, USA).

Raw FASTQ files were pre‐processed: after quality control by fastqc (i.e. reads with average base quality < 20 by means of an 8‐bp sliding window were discarded), adapters were removed. Sequences were then aligned to the GRCh37/hg19 human genome by bwa (https://github.com/lh3/bwa) and tophat2 (https://github.com/infphilo/tophat) tools. gatk (https://github.com/broadinstitute/gatk) and picard (https://github.com/broadinstitute/picard) were used for SNP variants calling, and annovar (https://github.com/WGLab/doc‐ANNOVAR) for SNP annotation. Case–control (AD vs. control) statistical association of SNPs was tested by plink (chi‐squared test). Different tools were used in combination to evaluate the variant's harmfulness: cadd (https://cadd.gs.washington.edu/), revel (https://sites.google.com/site/revelgenomics/), spliceai (https://ithub.com/Illumina/SpliceAI), pangolin (https://github.com/tkzeng/Pangolin), phylop (http://compgen.cshl.edu/phast/) and polyphen (http://genetics.bwh.harvard.edu/pph2/). Further, annotations for the R27S and D32G variants were extracted from the ClinVar (https://www.ncbi.nlm.nih.gov/clinvar/) and GnomAD (https://gnomad.broadinstitute.org/).

### Protein expression and purification

The genes encoding the R27S and D32G PSP variants and the R27S/D32G double variant were synthesized by Genscript (Piscataway, NJ, USA), employing the pET28_hPSP plasmid as a template [46]. The expression and purification process for wt PSP and its variants followed a previously published protocol [47]. Briefly, recombinant human PSP with a His‐tag was produced in *E. coli* BL21(DE3) cells and purified from the soluble fraction using a Talon Superflow resin (Cytiva, Marlborough, MA, USA). The purified protein was dialyzed against 25 mm Hepes and 100 mm NaCl, at pH 7.4, and then stored at −80 °C. The concentration of human PSP was calculated using an extinction coefficient of 11 460 m ^−1^·cm^−1^ at 280 nm, as determined by ProtParam (Expasy; http://www.expasy.org). Preparation of the enzymes phosphonucleotide phosphorylase (PNP) and serine acetyltransferase (SAT) followed the procedure reported previously [47].

### Far‐UV CD spectra and thermal stability

CD spectra of PSP variants were recorded using a Jasco 1500 spectropolarimeter (Jasco Co., Tokyo, Japan) with a Peltier temperature control system. Measurements were performed at 20 °C on 10 μm PSP solutions in buffer P (20 mm potassium phosphate buffer, 0.125 mm MgCl₂, pH 7.0), in a cuvette with a path length of 0.1 cm. Each spectrum was the average of three scans, corrected for buffer background.

Melting curves were recorded from 25 to 80 °C with detection at 210 nm, at 10 μm protein concentration in buffer P. Parameters included a heating rate of 5 °C·min^−1^, a 1 °C data pitch, a bandwidth of 1 nm and a digital integration time of 1 s. Data were fitted to Eqn (1) [48]:

(1) $$\theta =\theta_{0}+\frac{f}{1+e^{\frac{T-T_{m}}{k}}}$$

where *θ* is the ellipticity at 210 nm, *θ*_0_ is an offset, *f* is the amplitude of the thermal transition, *T* is the temperature in °C, *T*_m_ is the melting temperature and *k* is the slope of the transition.

### PSP activity assays

The kinetic parameters of the PSP variants were assessed by measuring l‐Ser production using a SAT‐coupled continuous assay, as described previously [47]. Assays were carried out in buffer H (50 mm Hepes, 100 mm KCl, pH 7) with the addition of 3 mm MgCl₂, 170 μm acetyl‐CoA, 0.5 mm 5,5‐dithio‐bis‐(2‐nitrobenzoic acid), 430 mU of SAT, and 3‐PS at concentrations ranging from 10 to 600 μm. Reaction mixtures were pre‐incubated at 37 °C in a microcuvette for 3 min before initiating the reaction by adding PSP at a final concentration of either 12 nm (for wt and R27S PSP) or 180 nm (for D32G and R27S/D32G PSP). Initial reaction velocities were calculated using the product extinction coefficient at pH 7.0 (14 100 m ^−1^·cm^−1^) [49, 50]. Kinetic parameters were derived by fitting the data to the Michaelis–Menten model (Eqn 2):

(2) $$v_{0}=\frac{V_{\max}\cdot \left[3‐\mathrm{PS}\right]}{K_{m}+\left[3‐\mathrm{PS}\right]}$$

where *v*_0_ is the initial velocity, *V*_max_ is the maximal velocity, [3‐PS] is the concentration of the substrate and *K*_m_ is the Michaelis constant.

To assess inhibition by l‐Ser, phosphate production was monitored using a PNP‐coupled assay [47]. Assays were conducted at 37 °C in buffer H containing 3 mm MgCl₂, 0.1 mm 7‐methyl‐6‐thioguanosine, 320 mU of bacterial PNP (Merck, Darmstadt, Germany), a 3‐PS concentration equal to *K*_m_, and l‐Ser from 0 to 10 mm. The 7‐methyl‐6‐thioguanosine concentration was estimated using an extinction coefficient of 32 000 m ^−1^·cm^−1^ at 331 nm, wheeas initial velocities were determined using a Δε of 11 200 m ^−1^·cm^−1^ at 360 nm [51]. The IC_50_ was calculated from the dependence of the initial rate on l‐Ser concentration using Eqn (3):
(3)

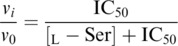

where *v*_0_ is the initial velocity in the absence of l‐Ser, *v*_*i*_ is the initial velocity at a given concentration of l‐Ser [l‐Ser], and IC_50_ is the concentration of l‐Ser producing 50% inhibition.

### *In vitro* pathway reconstruction

The *in vitro* reconstruction of the PP was carried out as previously described [8]. Enzyme concentrations for PHGDH, PSAT and PSP were matched to the intracellular levels observed in induced pluripotent stem cell‐derived astrocytes at 30 days of *in vitro* differentiation [18], whereas the concentration of substrates and cofactors was retrieved from literature data as reported previously [8]. In brief, all reactions were performed in buffer H supplemented with 0.53 mm 3‐PG, 0.3 mm NAD^+^, 2 mm l‐glutamate (l‐Glu), 0.3 mm MgCl₂, 0.1 mg·mL^−1^ BSA, 0.82 μm PHGDH, 1.14 μm PSAT and 0.12 μm PSP. The reaction was initiated by adding 3‐PG following a 5‐min pre‐incubation of the mixture at 37 °C. Then, 20 μL samples were taken at various time points and immediately mixed with 1% (w/v) trichloroacetic acid (TCA) to halt the reaction. To each sample, 100 μL of malachite green reagent (MAK308; Merck) were added, and the mixture was incubated for 30 min in the dark to allow full color development. Absorbance was then recorded at 620 nm using a HALO LED 96 microplate reader (Dynamica, Livingstone, UK). PHGDH and PSAT were expressed and purified as indicated previously [3, 4].

### SEC

SEC was performed using an Agilent 1260 HPLC system (Agilent), equipped with an Agilent AdvanceBio SEC 300 Å, 2.7 μm, 7.8 × 300 mm column (Agilent). The column was equilibrated with buffer H added at 3 mm MgCl_2_ and operated at a flow rate of 1 mL·min^−1^. The sample, containing wt PSP or its variants at either 0.1 or 2 mg·mL^−1^ was injected into the system and eluted at 25 °C. Elution profiles were monitored by recording the absorbance intensity at 280 nm using an Diode Array Detector 1260 Infinity II WR (Agilent). A set of molecular weight standards, including ferritin (440 kDa), alcohol dehydrogenase (140 kDa), conalbumin (75 kDa) and myoglobin (17 kDa), was used to calibrate the column, and retention times were converted into molecular masses using a standard calibration curve. Data were analyzed using Agilent GPC/SEC software for estimating the oligomeric state of the protein.

### *In silico* predictions

The three‐dimensional protein structures of both the wt PSP and its variants, along with their confidence scores, were computed using alphafold 3 (AF3) [20], accessed via the alphafold server. Visualization was performed using chimera x [52], where the Cα‐rmsd was also calculated using the ‘rmsd’ command. Of note, the N‐ and C‐termini of the predicted structures were not considered during the rmsd calculation because of their high flexibility. dynamut (https://doi.org/10.1093/nar/gky300) used via a web server (http://biosig.unimelb.edu.au/dynamut), was exploited to assess the impact of substitutions on protein dynamics and stability. The model of PSP bound to both products (phosphate and serine) and magnesium (PDB ID 6HYY, chain A) was used in the dynamut calculation.

### Cell culture studies

The open reading frame expression clone for human PSP NM_004577.4 (OmicsLink EX‐M0483‐M62 from GeneCopoeia, Rockville, MD, USA) was obtained by Tebu Bio (Le Perray‐en‐Yvelines, France). The construct contains a CMV promoter, a neomycin selectable marker, and expresses PSP with an AviTAG (a sequence encoding the 15 amino acid peptide tag GLNDIFEANKIEWHE) fused at the C‐terminus of the protein. The empty vector (EX‐NEG‐M62) was used as a control. The R27S and the D32G mutations were sequentially introduced into the PSP cDNA by nucleotides substitution at specific sites, using the QuikChange Lightning Site‐Directed mutagenesis kit (Agilent), the procedure provided by the supplier and the following couples of primers (substituted nucleotides are reported in bold and underlined): R27S forward: 5′‐GACAGCACGGTCATCAG **C** GAAGAAGGAATCGATGA‐3′; R27S reverse: 5′‐TCATCGATTCCTTCTTC **G** CTGATGACCGTGCTGTC‐3′; D32G forward: 5′‐ATCAGCGAAGAAGGAATCG **GA** GAGCTAGCCAAAATCTGTG‐3′; D32G reverse: 5′‐CACAGATTTTGGCTAGCTC **TC** CGATTCCTTCTTCGCTGAT‐3′. The presence of substitutions in the final vectors for the PSP variants expression was assessed by automated sequencing.

The U251 human glioblastoma cell line (ATCC; RRID:CVCL_0021) was chosen as a model system because it was previously shown to endogenously express the PP's enzymes [22]. Cells were maintained at 37 °C, in 5% CO_2_, in Dulbecco's modified Eagle's medium (Euroclone, Milan, Italy) supplemented with 10% fetal bovine serum, 1 mm sodium pyruvate, 2 mm l‐glutamine, 1% non‐essential amino acid, 1% penicillin–streptomycin and 1% amphotericin B. The medium was changed twice per week. Subculturing was performed by treatment with EDTA trypsin when cells reached 80–90% of confluence. All experiments were performed with mycoplasma‐free cells.

The expression of the wt and the R27S/D32G PSP AviTAG variants in the U251 human glioblastoma cell line was performed upon transfection using VibroFect technology and ViALL reagent (Vectorialis, Milan, Italy), which allows high transfection efficiency. Following the supplier's instructions, DNA (2.5 μg of the EX‐M0483‐M62 construct carrying the gene encoding wt or R27S/D32G variants, or the EX‐NEG control vector) was mixed with the VibroFect reagent in an Eppendorf tube and placed on the device for 10 s, producing ready‐to‐use stable nanoparticles that are added to the cells. After transfection, cell clones expressing stable levels of AviTAG PSP proteins were selected in Dulbecco's modified Eagle's medium with 0.4 μg·mL^−1^ G418 for approximately 3 weeks. Five U251 clones expressing wt PSP (U251^+wt^), five clones expressing the R27S/D32G variant (U251^+R27S/D32G^) and three clones transfected with the EX‐NEG‐M62 construct (U251^NEG^ controls) were isolated and stored for further studies.

For immunolocalization studies and confocal analyses, U251, U251^+wt^, U251^+R27S/D32G^ and U251^NEG^ cell clones were seeded onto rounded coverslips coated with 0.1% gelatine, in 24‐well plates. When cells reached 60–70% confluency on the coverslips, they were washed in phosphate‐buffered saline, fixed in 4% *p*‐formaldehyde for 10 min at room temperature and subjected to block‐permeation (in phosphate‐buffered saline, 0.1% Triton X‐100, 4% horse serum) for 20 min at room temperature. Coverslips were then incubated overnight at 4 °C with the following antibodies: mouse monoclonal anti‐AviTAG (dilution 1 : 250; GenScript) and rabbit anti‐PHGDH (dilution 1 : 1000, HPA024031; Sigma‐Aldrich, St Louis, MO, USA) or mouse anti‐PSAT (dilution 1 : 250, H00029968‐A01; Abnova, Taipei, Taiwan), and then in goat anti‐mouse AlexaFluor 546 or 488, and goat anti‐rabbit AlexaFluor 488 antibodies, for 1 h at room temperature. For the double staining AviTAG–PSAT, two subsequent incubations were performed: with the anti‐AviTAG and the anti‐mouse AlexaFluor 546 antibodies, and then with the anti‐PSAT and the goat anti‐mouse AlexaFluor 488 antibodies. After extensive washing, coverslips were mounted by using a 4´,6‐diamidino‐2‐phenylindole‐containing mounting medium to allow nuclei staining. Images were acquired using A1R laser scanning confocal microscope (Nikon, Tokyo, Japan) with a 60× (NA 1.4) oil immersion objective. 4´,6‐Diamidino‐2‐phenylindole, AlexaFluor 488 and 546 were excited using a 405, 488 and 543 nm laser light, respectively, and the emitted signals were collected in the 425–475, 500–550 and 570–620 nm range, respectively. For distribution and colocalization analyses, 10 different fiedls were acquired for each coverslip. After acquisition, images were deconvoluted using the Richardson–Lucy algorithm in the deconvolution module of nis‐elements acquisition software (Nikon).

Image analysis was performed using fiji (https://imagej.net/software/fiji). The colocalization and correlation of the detected immunofluorescence signals were analyzed using the biop jacop plugin (by Fabrice P. Cordelières, Bordeaux Imaging Center, France; revamped by the BIOP; https://github.com/BIOP/ijp‐jacop‐b). Regions of interest (ROI) containing single cells (*n* = 30 and 40 for U251^+wt^ and U251^+R27S/D32G^, respectively) were selected and processed by applying the Otsu threshold for both the red (AviTAG) and the green (endogenous PP's proteins) channels. The total density of signals within selections (the RawIntDen value, corresponding to the sum of the values of the pixels in the 2D selection for each channel) and the areas of the selected ROIs (identifying nuclei or cells) were determined using the measure tool in the ROI manager. Approximating cells and nuclei to spheres, the radius *r* was calculated based on the area of the corresponding selections. If the 2D image represents an equatorial section, the ratio between the section area (*A* section) and the volume of the sphere (*V* sphere) is given by Eqns ((4), (5), (6)):

(4) $$C=\frac{A_{\text{section}}}{V_{\text{sphere}}}$$

where *A*_section_ = π*r* ^2^ and *V*_sphere_ = 4/3π*r* ^3^; therefore

(5) $$C=\frac{r^{2}}{\frac{4}{3}r^{3}}=\frac{3}{4r}$$

and

(6) ρ=RawIntDen*C

### Cellular stability

To evaluate the cellular stability of wt and R27S/D32G PSP variants, U251^+wt^, U251^+R27S/D32G^ and U251^NEG^ cell clones were seeded into six‐well plates (2.5 × 10^5^ cells per well) and treated for up to 48 h with cycloheximide (100 μg·mL^−1^; Sigma‐Aldrich), a potent inhibitor of protein synthesis. Changes in the cellular levels were evaluated by western blot and densitometric analysis of samples collected at different times (0, 6, 24 and 48 h). Data were fit to a single exponential decay equation to estimate the half‐life. For statistical significance, three different clones for each cell type were analyzed.

For Western blot analysis, cells were resuspended in sample buffer (12.5 mm Tris–HCl, pH 6.7, 3% SDS, 5% glycerol and 62.5 mm dithiothreitol) to obtain 5000 cells·μL^−1^ and 20 μL of each sample was subjected to SDS/PAGE. Proteins were then transferred onto a poly(vinylidene difluoride) membrane and saturation of aspecific sites was performed by overnight incubation at 4 °C with 4% dried milk in Tris‐buffered saline (TBS), pH 8.0, with added 0.1% Tween‐20. Membranes were then incubated at room temperature for 1.5 h with rabbit anti‐PSP (dilution 1 : 1000; PA5‐22003; Invitrogen, Waltham, MA, USA) or mouse anti‐α‐tubulin antibodies (dilution 1 : 4000; T6074; Sigma‐Aldrich) diluted in TBS, pH 8.0, with added 2% dried milk and 0.05% Tween 20. After extensive washing, the membranes were incubated for 1 h at room temperature with goat anti‐rabbit IgG (dilution 1 : 20 000; A32735, Alexa‐Fluor Plus 800; Invitrogen) and goat anti‐mouse IgG (dilution 1 : 10 000; A32729, Alexa‐Fluor Plus 680; Invitrogen) diluted in TBS, pH 8.0, and 0.05% Tween 20. Membranes were analyzed by an Odyssey Fc Imaging system and image studio (LI‐COR Biosciences, Lincoln, Nebraska, USA): the intensity of the bands corresponding to the ectopically or endogenously expressed PSP (detected by the anti‐PSP antibody as two distinct bands, as a result of the difference in the molecular mass) was normalized by the tubulin signal. The PSP variants content in the samples was determined using known amounts of the purified recombinant protein and was related to the number of cells corresponding to the analyzed sample.

### Enantiomeric HPLC

The selected U251^+wt^, U251^+R27S/D32G^ and control U251^NEG^ cell clones were homogenized in 50–60 μL of 0.2 m TCA to extract intracellular components and precipitate proteins. The suspension was sonicated in an ultrasound bath for 20 min and centrifuged at 16 000 ***g*** for 30 min at 4 °C. The supernatant was stored for HPLC analysis, whereas the protein pellets were solubilized in 1% SDS, sonicated in an ultrasound bath for 20 min, clarified by centrifugation, and used for total protein quantification with the Bradford assay (500‐0205; Bio‐Rad, Hercules, CA, USA).

Next, 5 μL of TCA‐sample were neutralized with 4.5 μL of NaOH and subjected to pre‐column derivatization with 10 μL of a solution containing 20 mg of *o*‐phthaldialdehyde and 10 mg of *N*‐acetyl l‐cysteine dissolved in 2 mL of 50% methanol. The separation of the derivative diastereoisomers was carried out under isocratic conditions (1 mL·min^−1^ flow rate) using a HPLC system (PU2089, FP2020, AS2059) (Jasco Co.) on a Symmetry C8 reversed‐phase column (5 μm, 4.6 × 250 mm; Waters, Milano, Italy). Then, 0.1 m sodium acetate buffer, pH 6.2, with added 1% tetrahydrofuran was used as the mobile phase, and a washing step with 15 mL of 0.1 m sodium acetate buffer at pH 6.2, 47% acetonitrile and 3% tetrahydrofuran was performed after each run. Identification and quantification of amino acids were based on retention times and peak areas compared to those obtained with external pure standards. As further control, the d‐Ser content was confirmed by incubating each sample with the M213R variant of the d‐amino acids degrading enzyme *Rg*DAAO for 4 h at 30 °C before derivatization [53]. The amino acid content expressed in nmol was normalized based on the amount (mg) of total proteins.

## Conflicts of interest

The authors declare that they have no conflicts of interest.

## Author contributions

LP and BC designed the project and conceptualized the approach. LP, BC, AP and SB supervised the project. IA performed the bioinformatic analyses. VBC, FM and ODB performed the biochemical characterization of recombinant PSP variants. ODB and VBC performed the *in silico* predictions. VR performed the cellular stability studies. ZM performed the chromatographic analyses. SS performed the cellular studies. All of the authors performed data curation and wrote the original draft. VBC, FM, ODB, ZM, VR and SS prepared illustrations. All authors have read and approved the final version of the manuscript submitted for publication.

## Supporting information


**Fig. S1.** Analysis of human PSP degradation rate.
**Fig. S2.** Half‐life analysis of endogenous and Avi‐tagged PSP variants in U251 cell clones.
**Table S1.** Heterozygous variants in analyzed AD and CTR samples for D32G and R27S PSP variants.
**Table S2.** The nuclear vs. cytosolic distribution of the overexpressed wild‐type and R27S/D32G PSP variants.

## Data Availability

The data that support the findings of this study are available from the corresponding authors upon reasonable request.
