# Supplementary material for: A phosphoserine phosphatase variant present in the brain of Alzheimer's disease patients favors nuclear mistargeting
Source: FEBS J. 2025 Jul 18;292(18):4955–74. doi: 10.1111/febs.70169 (PMC12443467; doi:10.1111/febs.70169)
Supplement: Supplementary file 1 — Fig. S1. Analysis of human PSP degradation rate. Fig. S2. Half‐life analysis of endogenous and Avi‐tagged PSP variants in U251 cell clones. Table S1. Heterozygous variants in analyzed AD and CTR samples for D32G and R27S PSP variants. Table S2. The nuclear vs. cytosolic distribution of the overexpressed wild‐type and R27S/D32G PSP variants. [file FEBS-292-4955-s001.pdf]

SUPPLEMENTAL FIGURES & TABLES

A VARIANT PHOSPHOSERINE PHOSPHATASE IN THE BRAIN OF ALZHEIMER'S DISEASE PATIENTS FAVOURS NUCLEAR MISTARGETING

Silvia Sacchi[1*], Valeria Buoli Comani[2*], Ivan Arisi[3,4], Francesco Marchesani[5], Valentina Rabattoni[1], Omar De Bei[5], Zoraide Motta[1], Alessio Peracchi[6], Stefano Bruno[2], Loredano Pollegioni[1§], Barbara Campanini[2§]

[1] The Protein Factory 2.0, Dept of Biotechnology and Life Sciences, University of Insubria, Varese, Italy

[2] Dept of Food and Drug, University of Parma, Italy

[3] European Brain Research Institute (EBRI) Rita Levi-Montalcini, Rome, Italy

[4] Institute of Translational Pharmacology, National Research Council, Rome, Italy

[5] Dept of Medicine and Surgery, University of Parma, Italy

[6] Dept of Chemistry, Life Sciences and Environmental Sustainability, University of Parma, Italy

*these two authors contributed equally to the work

§correspondence to: loredano.pollegioni@uninsubria.it; barbara.campanini@unipr.it

Table S1. Heterozygous variants in analyzed AD and CTR samples for D32G and R27S PSP variants.

		**CTR samples variants**										**AD samples variants**										
	**Sex**	**F**	**F**	**F**	**F**	**F**	**M**	**M**	**M**	**M**	**M**	**F**	**F**	**F**	**F**	**F**	**F**	**M**	**M**	**M**	**M**	**M**
	**Diagnosis**	**CTR**	**CTR**	**CTR**	**CTR**	**CTR**	**CTR**	**CTR**	**CTR**	**CTR**	**CTR**	**AD**	**AD**	**AD**	**AD**	**AD**	**AD**	**AD**	**AD**	**AD**	**AD**	**AD**
**SNP ID**	**Mutation**																					
rs78599516	7-56088811-T-C (D32G)	.	.	.	.	.	.	.	.	.	.	.	0/1	.	0/1	.	0/1	.	.	.	0/1	.
rs74445297	7-56088825-T-A (R27S)	.	.	.	.	.	.	.	.	.	.	.	.	.	0/1	.	0/1	.	.	.	0/1	.
rs202027697	7-56088786-A-T	.	.	.	.	.	.	.	.	.	.	.	.	.	0/1	.	0/1	.	.	.	.	.
rs199851385	7-56088789-G-A	.	.	.	.	.	.	.	.	.	.	.	.	.	0/1	.	0/1	.	.	.	.	.

Table S2. The nuclear vs. cytosolic distribution of the overexpressed wild-type and R27S/D32G PSP variants (as well as of the endogenous PHGDH and PSAT enzymes) in selected cell clones was evaluated by immunofluorescence and confocal analysis on single sections including the nucleus. The reported values represent the portion of the volumetric density ρ (the total amount of signal normalized by the estimated volume of the cellular compartments), detected in the nucleus and the cytoplasm. Data represent mean values ± SEM.

	**AviTAG**		**PHGDH**		**PSAT**	
**Distribution (% ρ)**	$U251^{+wt}$	$U251^{+R27S/D32G}$	$U251^{+wt}$	$U251^{+R27S/D32G}$	$U251^{+wt}$	$U251^{+R27S/D32G}$
**Nucleus**	23.6±1.2	28.6±1.3	14.0±1.3	20.4±1.0	22.3±1.8	22.9±2.4
**Cytoplasm**	76.4±1.2	71.4±1.3	86.0±1.3	79.6±1.0	77.7±1.8	77.1±2.4

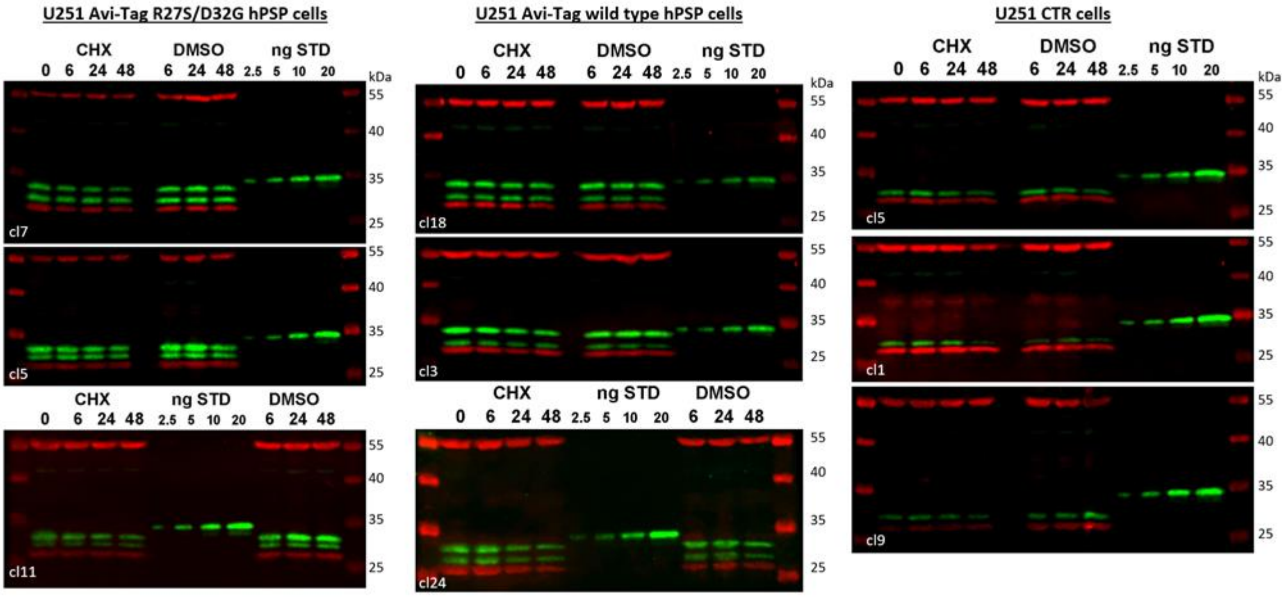

Figure S1. Analysis of human PSP degradation rate. U251 cell clones stably expressing Avi-tagged R27S/D32G or wild-type PSP and control cells (transfected with empty pBUDNeo vector) were treated for up to 48 hours with 100 µg mL^{-1} CHX or DMSO as a control (n=3). The R27S/D32G variant showed a modest reduction in half-life compared to Avi-tagged or endogenous wild-type PSP: after 48 hours of CHX treatment, approximately 50% of the variant protein remains in the cell clones, compared to an average of 71±6% for the wild-type one (Avi-tagged/endogenous). The signal of the primary antibody against PSP is shown in green while the signal detected using the anti-tubulin antibody, used for signal normalization, is shown in red. Recombinant human PSP (27.2 kDa, STD) was used as a positive control.

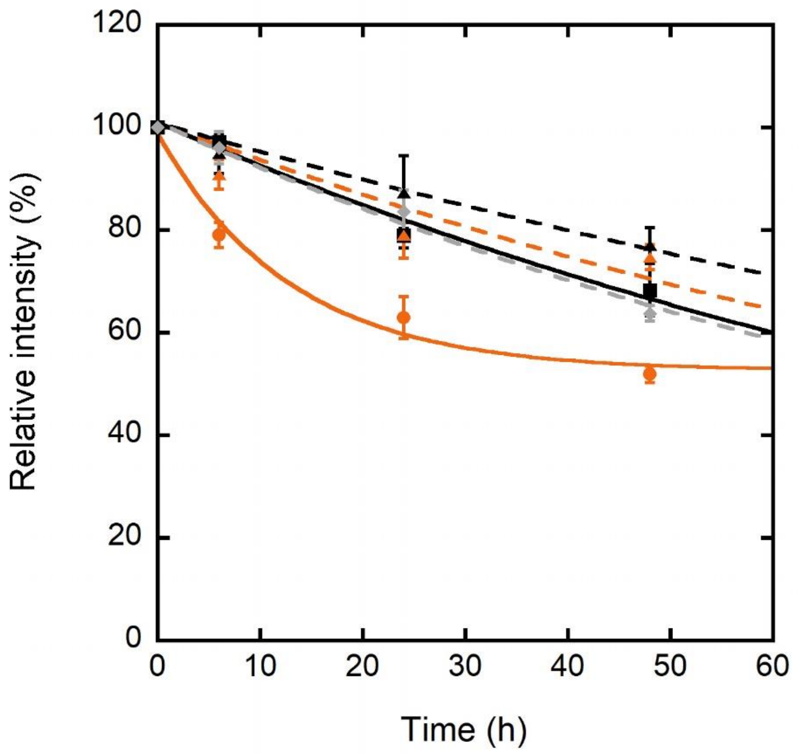

Figure S2. Half-life analysis of endogenous and Avi-tagged PSP variants in U251 cell clones. The fitting of experimental data was carried out using a single exponential decay. The level of endogenous PSP was analyzed both in control cells (NEG, transfected with empty pBUDNeo vector, dashed grey line) and in the cell clones expressing the Avi-tagged PSP variants (wt: dashed black line; R27S/D32G: dashed orange line). The level of Avi-tagged PSP wt is indicated in black continuous line, and of R27S/D32G in orange continuous line. Values are the mean ± SD (n = 3), normalized to tubulin, and expressed relatively to untreated cells (time 0).
